# Effects of Hypoxia Stress on Growth, Root Respiration, and Metabolism of *Phyllostachys praecox*

**DOI:** 10.3390/life12060808

**Published:** 2022-05-29

**Authors:** Jiawei Ma, Gul Rukh, Zhongqiang Ruan, Xiaocui Xie, Zhengqian Ye, Dan Liu

**Affiliations:** 1 Key Laboratory of Soil Contamination Bioremediation of Zhejiang Province, Zhejiang A & F University, Lin’an 311300, China; chaoticant@outlook.com (J.M.); ruanzhognqiang@163.com (Z.R.); xxc3043862950@163.com (X.X.); yezhq@zafu.edu.cn (Z.Y.); 2 The Nurturing Station for the State Key Laboratory of Subtropical Silviculture, Zhejiang A & F University, Lin’an 311300, China; 3 Department of Chemistry, The Islamia College University Peshawar, Peshawar 25120, Pakistan; gulrukhshafi@gmail.com

**Keywords:** hypoxia stress, physiology and biochemistry, metabonomics, *Phyllostachys praecox*

## Abstract

Hypoxia affects plant growth, hormone content, various enzyme activities, cell structure, peroxide production, and metabolic level, therefore reducing crop yield. This study assessed the physiological, biochemical, and metabolic characteristics of *Phyllostachys praecox*. Results revealed that hypoxia stress treatment significantly inhibited plant growth. Leaf chlorophyll contents was initially improved and then reduced with plant growth time. Under hypoxia stress, the root activity significantly was reduced, leading to the decrease in the nutrient absorption and transport. Yet, with low oxygen concentration, the contents of ethanol, acetaldehyde, and lactic acid were improved. With hypoxia stress, phospholipids and amino acids were the main metabolites of *Phyllostachys praecox*. Glycosphospholipid metabolism is the key pathway in responding to hypoxia stress significantly (*p* < 0.05), and lysophosphatidlycholine (lysoPC) and phosphatidylcholines (PC) in the metabolites of this metabolic pathway were significantly enhanced. Our study reveals the mechanism of *Phyllostachys praecox* cell membrane responding to hypoxia stress based on molecular level. This is conducive to finding targeted solutions to improve the productivity of *Phyllostachys praecox* to better optimize a mulching approach in the bamboo forest.

## 1. Introduction

Since the soil oxygen environment is positively related to soil nutrients, its aeration is important factor to affect plants growth [1,2]. Indeed, hypoxia stress, a kind of non-habitat stress, governs insufficient oxygen supply in the rhizosphere due to flood, flooding, and improper irrigation [3,4], leading to an adverse impact on crop growth, yield, and quality [5]. This is mainly attributed to the fact that crop roots rely on energy generated by soil oxygen respiration to maintain various physiological activities [6]. Intensive management with organic material mulching is a special mode of bamboo industry, especially in the Zhejiang province of China [7]. This management is mulched with rice husk or straw with a thickness of 30 cm, largely reducing the oxygen content of the soil layer to 7% during intensive management of the *Phyllostachys praecox* forest [8]. Despite that aeration of covered *Phyllostachys praecox* can be positively against this effect [9], there were few reports on the effect of oxygen deficiency on growth and response of *Phyllostachys praecox*.

Hypoxic stress affects plant growth via altering normal physiological metabolism. For example, this stress decreases accumulation of plant biomass, hormone content, various enzyme activities, cell structure, and peroxide production, therefore reducing metabolic level and plant growth [10,11]. It also induces other hazards, such as inhibiting the activity of aerobic microorganisms and slowing down decomposition of organic matter in soils, therefore reducing plant available nutrients but increasing toxic substances [12,13]. With the stress, the plant cell fermentation produces free acids, such as lactic, butyric, and propionic acid, leading to cytoplasmic acidification [14]. This process further induces plant to form anti hypoxia mechanisms, such as formation of ventilatory tissue and accelerated decomposition of toxic substances [15,16]. As being revealed by previous studies, hypoxic stress inhibited aerobic respiration of maize and wheat via weaking anaerobic respiration metabolism and anaerobic respiration. However, this mechanism in response to hypoxic stress significantly varies with plant species [17,18].

Thus, it still needs to further explore both physiological-biochemical and molecular level mechanism in responding to hypoxia environment during plant growth. This study used *Phyllostachys praecox* to analyze the effects of different rhizosphere hypoxia treatments on its growth, respiratory products, respiratory key enzymes, nutrient absorption, and metabolism of *Phyllostachys praecox* seedlings. Our findings are to provide a theoretical basis for exploring the stress resistance mechanism of bamboo under hypoxia stress.

## 2. Materials and Methods

### 2.1. Experimental Materials

The *Phyllostachys praecox* seedlings were collected from annual bamboo cultivation in September 2021. These seedlings were shifted to a water training forest and then selected for the hydroponics experiment in December 2021.

### 2.2. Hydroponics Experiment

The culture was carried out in 1/2 Yoshida nutrient solution in 5 L black drum (200 mm in diameter, 300 mm in height). Each container was filled with 4 L of nutrient solution. The 6 water-cultured seedlings were cultured in each pot with 3 repetitions. The nutrient solution was replaced after every 4 days. The seedlings were incubated in a light incubator for 18 h at 25 °C and dark culture for 6 h at 20 °C. The 5 mL of solution was added in every 4 L of water, and pH was adjusted to 5.2 value with 1 mol/L NaOH or 1 mol/L HCl. The composition of nutrients solution was illustrated in previous study [19].

The test was divided into 4 treatments (T) and set as follows: T1 was ventilation pump ventilation, oxygen concentration about 8 mg/L; T2 was oxygen concentration about 6 mg/L (ventilation was a dual channel of nitrogen and oxygen, and dissolved oxygen concentration in water was controlled to be about 6 mg/L); T3 was oxygen concentration about 4 mg/L; and T4 was oxygen concentration about 2 mg/L. The dissolved oxygen controller was equipped with a dissolved oxygen measuring instrument, which could measure oxygen concentration in the solution in real time and automatically adjust the ventilation rate. According to the pre-experiment, the ventilation rate was 200 mL/min. To ensure the consistency of dissolved oxygen in the nutrient solution before treatment, the nutrient solution with nitrogen was filled to remove oxygen for 15 min. The mass concentration of dissolved oxygen in the nutrient solution before treatment was 2.0 mg/L, which was connected to ventilation device for stabilization.

According to the pre-culture experiment, it was observed that leaves began to curl after two weeks of hydroponic culture (14 days). The culture time was set as 12 days, and the sampling was performed on 0, 3, 5, 7 and 12 days, respectively. Chlorophyll was measured before sampling, and respiration rate of roots was measured; fresh samples were collected for determination of respiratory enzymes, respiratory products, malondialdehyde, ATP, and other indicators. The plants were harvested after 12 days of the experiment, and the net biomass was measured. The root and leaf samples were collected for green killing and drying for nutrient determination. The fresh samples were collected for liquid nitrogen collection for non-targeted metabolomic analysis.

### 2.3. Physiological and Elemental Determination

#### 2.3.1. Determination of Leaf Chlorophyll Contents (SPAD)

The determination of leaf chlorophyll contents was according to Pagola method [20] using SPAD 502. The relative chlorophyll content was measured: two-thirds of fresh leaves from leaf margin and three leaves were randomly determined for each plant. The SPAD parameters were measured in vitro from leaves of plants. Each leaf was repeated for 3 times.

#### 2.3.2. Determination of Malondialdehyde (MDA)

MDA concentrations were determined according to Chen method [21]. The 1 g fresh leaf samples were grounded with 10 mL 0.1% (*w*/*v*) trichloroacetic acid (TCA) in ice-bath conditions. The homogenate was centrifuged at 4000 r/min for 20 min, and supernatant was utilized for next chromogenic reaction with thiobarbituric acid (TBA). This test determined MDA as a product of lipid peroxidation, which was used for measurement of lipid peroxidation in samples [22].

#### 2.3.3. Determination of Plant Root Vitality and Respiratory Products

The roots were washed using distilled water, and its activity was measured with TTC method [23]. The fresh leaf samples of 0.5 g were homogenized with 10 mL of 0.4% TTC solution and phosphoric acid buffer, fully immersing the root in the solution. The roots were kept in dark at 37 °C for 1 h, and then, 2 mL of 1 mol/L sulfuric acid was added to stop the reaction. The roots were removed from the dark and grounded with ethyl acetate 3–4 mL and a small amount of quartz sand to get TTF. The red extraction solution was transferred to test tube. The residue was washed with small amounts of ethyl acetate, and transferred in test tube. The volume was finally adjusted to 10 mL to compare the color using spectrophotometer at 485 nm. The OD was read out with a blank as reference, and standard curve was checked. The reduction amount of tetrazolium was calculated. The extraction and determination of acetaldehyde, ethanol, and lactic acid were carried out according to methods of Good et al. [24] and Bergmeyer method [25].

#### 2.3.4. Element Analysis in Pants

Plant samples were dried at 70 °C for 72 h. The oven-dried plant parts were passed through 0.1 mm nylon sieve for N, P, K, Na, Ca, and Mg analysis. Total N was determined by Kjeldahl distillation titration after the solution to be tested was boiled with H_2_SO_4_-H_2_O_2_. Plant P in the test solution was determined by molybdenum antimony anti colorimetry. K, Na, Ca, and Mg were digested using the mixtures of HNO_3_/HClO_4_, and the supernatant was extracted and analyzed by flame atomic absorption spectrometry (FAAS, PerkinElmer AA800, Los Angeles, CA, USA).

#### 2.3.5. Metabolic Profiling

Metabolites were detected using an LC-MS analysis platform (UHPLC -Q Exactive HF-X, Thermo Fisher Scientific, Waltham, MA, USA) at Majorbio (Shanghai, China). Chromatographic conditions: The chromatographic column was ACQUITY UPLC HSS T3 (100 mm × 2.1 mm I.D., 1.8 µm; Waters, Milford, MA, USA); mobile phase A consisted of 95% water +5% acetonitrile (containing 0.1% formic acid), and mobile phase B consisted of 47.5% acetonitrile +47.5% isopropanol +5% water (containing 0.1% formic acid). The injection volume was 2 μL, and the column temperature was 40 °C. The samples were ionized by electrospray ionization, and the positive and negative ion scanning modes were used to collect the mass spectrum signals.

### 2.4. Statistical Analysis

Microsoft Excel 2016 (Microsoft, Redmond, WA, USA) and SPSS 24.0 (SPSS Inc., Chicago, IL, USA) software were used for data analysis, and Origin 8.5 (OriginLab Corp., Northampton, MA, USA) software was used for drawing statistical charts. Data were tested with a significance level at *p* < 0.05 using one-way analysis of variance (ANOVA).

Metabolome data were imported into ProgenesisQI (Waters Corporation, Milford, MA, USA) to identify metabolites. PCA analysis was performed to reduce the dimensionality of the multidimensional data. We compared their mass spectra with the mass-to-charge ratio (*m*/*z*) offered in the free online databases, including MEILIN (http://metlin.scripps.edu/, accessed on 1 December 2020) and KEGG (http://www.genome.jp.kegg/, accessed on 1 December 2020). Finally, the metabolic pathway analysis module in MetaboAnalyst 3.0 was applied to explore the links between responsive metabolites and potential metabolic pathways.

## 3. Result

### 3.1. Effects of Hypoxia Stress on Growth

#### 3.1.1. Changes of Crop Growth and Chlorophyll Content

**Figure 1:**
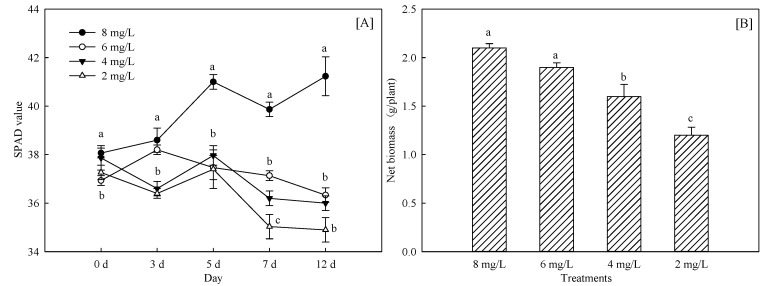
Effects of hypoxia stress on relative growth (**A**) and SPAD value (**B**) of *Phyllostachys praecox*. Data points and error bars represent mean ± S.D. of three replicates (*n* = 3). Different letters indicate a significant difference between different treatments in the same period (*p* < 0.05).

The treatments of hypoxia stress significantly inhibited growth of *Phyllostachys praecox* (Figure 1). The maximum reduction value of biomass was 42.8% with 2 mg/L treatment, followed by 4 mg/L treatment and compared with control. The SPAD value initially improved and then fell with time in the whole culture cycle. There was no significant difference after 3 days, but there was significant variation between different treatments with extension of culture time to 7 days. Maximum reduction value in biomass was 20.0% with 2 mg/L treatment and 6 mg/L. The decrease in net biomass was also observed in 4 mg/L treatment, but there was no significant variation after 12 days.

#### 3.1.2. Effects of Hypoxia Stress on Nutrient Absorption of Phyllostachys Praecox

**Figure 2:**
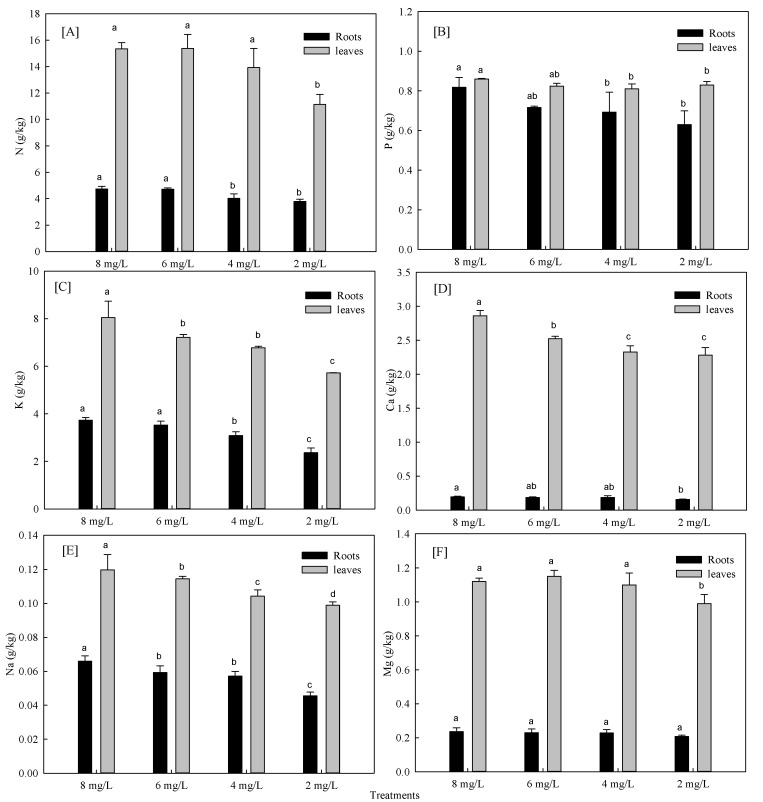
Effects of hypoxia stress on nutrient N (**A**), P (**B**), K (**C**), Ca (**D**), Na (**E**) and Mg (**F**) uptake of *Phyllostachys praecox*. Data points and error bars represent mean ± S.D. of three replicates (*n* = 3). Different letters indicate significant difference between different treatments in the same period (*p* < 0.05).

The hypoxia stress significantly decreased absorption and transport of nutrients. The nutrient content in stems and leaves per unit mass was reduced under hypoxia stress. As revealed in Figure 2, root nutrient absorption was diminished with hypoxia stress. After 12 days of hypoxia treatment, the content of different ions decreased significantly compared with control, in which K^+^ decreased up to 37.0%, followed by Na^+^ with 2 mg/L. The most influential nutrients were N, P, and K under 4 mg/L hypoxia treatment. The nutrient content of leaves varied with treatments. Yet, N and K were the most significant, and their decline ratio was lower than that of roots in hypoxia stress of 2 mg/L. This indicates that root system was greatly affected by energy and other physiological aspects in terms of nutrient absorption. Different nutrients in both leaves and roots were affected by hypoxia stress. The reduction rate of K^+^ was the largest, while other nutrients varied.

#### 3.1.3. Effects of Hypoxia Stress on MDA in Roots

MDA reflects the degree of tissue peroxidation damage. Since MAD accumulation causes some damage to membrane and cells, its content can be used as one of the indicators to investigate the severity of cell stress. As being observed in Table 1, MDA content was improved with increasing the degree of hypoxia stress and its treatment time. The MDA increased by 2.65 times in treatment of 2 mg/L. The MDA was raised by 2.1 times in treatment of 4 mg/L treatment and enhanced by 55% in treatment of 6 mg/L compared with control. MDA increased with reducing oxygen concentration.

### 3.2. Effects of Hypoxia Stress on Root Activity and Respiratory Products

**Figure 3:**
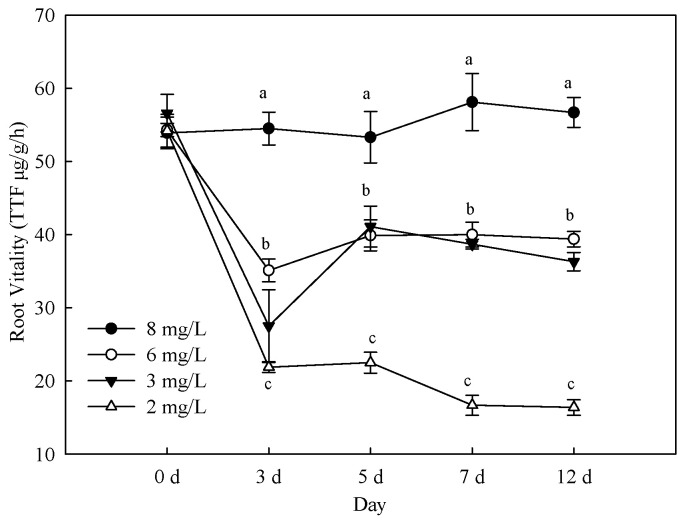
Changes of root activity of *Phyllostachys praecox* during hypoxia stress. Data points and error bars represent mean ± S.D. of three replicates (*n* = 3). Different letters indicate significant difference between different treatments in the same period (*p* < 0.05).

Figure 3 exhibits the effects of different hypoxia treatments on root respiration rate. The activity of seedling root system was reduced with reduction of dissolved oxygen content. The root respiration rate initially decreased and then enhanced with time of hypoxia treatment but maintained a low respiration rate at 2 mg/L. Minimum value of lowest root activity was 70.0% with 2 mg/L treatment compared with control on the 12th day. The root respiration rate was significantly reduced than other treatment in 2 mg/L treatment (*p* < 0.05). The root activity was significantly lower than control in 4 mg/L and 6 mg/L treatment. The hypoxia stress affected plant TCA cycle, intermediate product accumulation, and anaerobic respiration. As can be observed in Table 2, all contents of ethanol, acetaldehyde, and lactic acid was enhanced with reducing oxygen concentration.

### 3.3. Metabolome Analysis

#### 3.3.1. PCA Analysis

**Figure 4:**
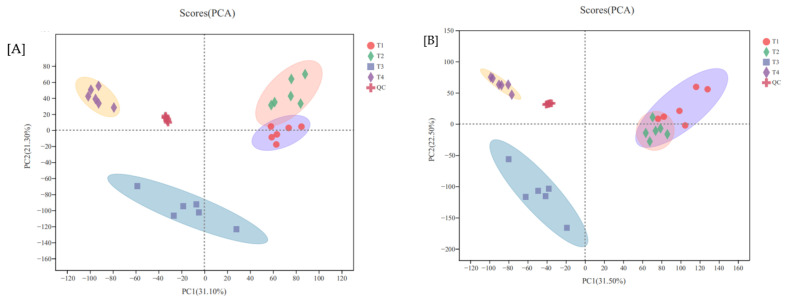
Cation PCA (**A**) and anionic PCA (**B**) diagram of metabolites in leaves of *Phyllostachys praecox* under hypoxia stress.

As revealed in Figure 4 (cation), PC1 elucidated 31.1% of total variance, and PC2 exhibited 21.3% of total variance. Anionic PCA diagram showed that PC1 explained 31.5% of the total variance, and PC2 illustrated 22.5% of the total variance.

The four treatments were obviously separated in space. Discrimination between treatments 3 and 4 and 1 and 2 was large, while distinction between treatments 1 and 2 was weak, especially for anions. The QC could correlate, showing that data quality and the repeatability was reliable, and there are obvious variations among the test sample groups.

#### 3.3.2. Metabolites

According to KEGG compound annotation classification (Level II), we detected the main categories of metabolites, including phospholipids (48 species), amino acids (16 species), eicosanoids (11 species), monosaccharides (9 species), nucleotides (9 species), fatty acids (8 species), carboxylic acids (8 species), vitamins (6 species), steroid hormones (5 species), and amines (4 species), etc.

The differential metabolites were screened according to (FC > 2, or <0.5, VIP > 1, and *p* value < 0.05). The first column in Table 3 was the ion source mode; the second column total number represented the number of names of the identified differential metabolites; and the number of ion peaks meeting the differential screening conditions is shown in parentheses.

Table 3 showed that there were 41 differential metabolites that met the differential screening conditions, of which 23 were up-regulated, and 18 were down-regulated in group A. There were 60 differential metabolites that met the differential screening conditions, of which 56 were up-regulated, and 4 were down-regulated in group B. There were 38 differential metabolites that met the criteria of differential screening conditions, of which 26 were up-regulated, and 12 were down-regulated in group C. Among them, there were three differential metabolites in three differential groups. The specific differential metabolites of each differential group were 8 (a), 74 (b), and 72 (c), respectively. The screened differential metabolites provided effective data supporting for further analysis.

#### 3.3.3. Metabolic Pathway

According to statistics of the number of compounds annotated to the KEGG metabolic pathway (II), the main metabolic pathways included amino acid metabolism (135), lipid metabolism (91), carbohydrate metabolism (53), nucleotide metabolism (29), membrane transport (27), signal transduction (16), and energy metabolism (10), etc.

**Figure 5:**
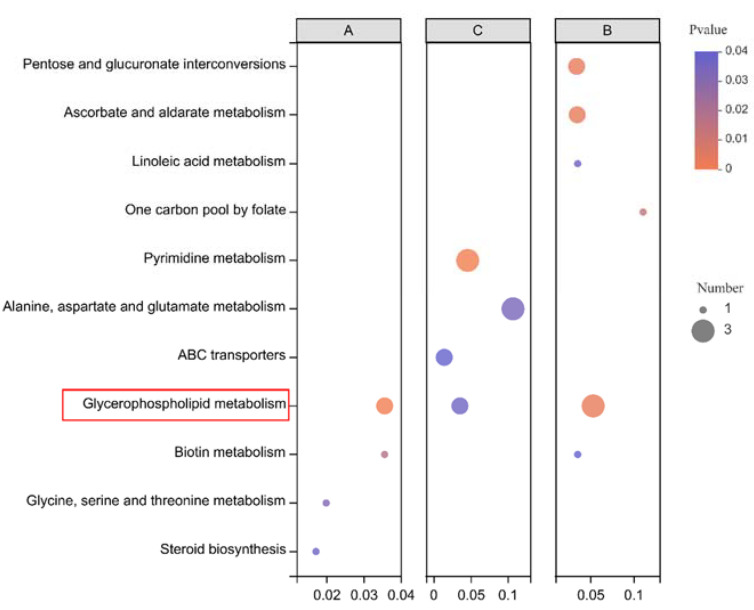
Pathway identification among A (T1 vs. T2), B (T1 vs. T3), and C (T1 vs. T4). The bubble charts show the enrichment of metabolites in the affected pathways (*p* < 0.05). The *y*-axis label provides the pathway names, and the *x*-axis label indicates the comparison group. Each circle represents a metabolic pathway, and the color and size of circle represent the results of metabolite enrichment and topology analysis, respectively.

The functional pathways of the three groups of differential metabolic sets were annotated and enriched to obtain the differential metabolic pathways (Figure 5). The common metabolic pathway of the three groups (*p* < 0.05) was glycophospholipid metabolism, indicating that lipid metabolism is the key pathway to deal with hypoxia stress.

## 4. Discussions

Since root respiration was disturbed by hypoxia stress, plant cells carried on a series of physiological, biochemical, and morphological alteration, therefore inhibiting plant growth and development [26,27]. However, this effect depends on the various treatment. As illustrated in this study, whole-plant biomass was largely reduced by treatment of 2 mg/L oxygen concentration. In particular, this inhibitory effect of hypoxia treatment on plants was significantly enhanced during key root growth period.

Indeed, root respiration rate indicates activity of exchange between plants and in vitro. It is not only an important indicator of root activity but also an important link affecting uptake of plant mineral elements [28]. This is attributed to aerobic respiration, and its circulation is disturbed. Once being exposed to hypoxia, concentration of oxygen in rhizosphere decreases, while available oxygen is reduced, diminishing root respiration. This experiment reveals that hypoxia treatment significantly reduced root respiration rate. After 2 days of hypoxia treatment, the root respiration decreased but increased after 5 days and tended to a stable state. This may be a stress response of plants to hypoxia. A series of physiological and biochemical reactions occur synchronously and adjust to a new equilibrium point, which is the strategy of plants in dealing with hypoxia treatment. The root respiration was weakened to its nutrient absorption, recuing aboveground nutrient content and therefore inhibiting plant growth [29].

Mitochondria and chloroplasts are organelles for energy conversion in cells. Their inner membrane is not only the carrier of energy conversion but also the chemical reaction site of mitochondrial oxidative phosphorylation and chloroplast photosynthesis [30,31]. There is an electron transport chain (respiratory chain) related to oxidative phosphorylation on the inner membrane of mitochondria. The electron respiratory chain on the inner membrane of mitochondria first stagnates to produce ATP, and the tricarboxylic acid cycle is blocked, accumulating many intermediate products, such as ethanol, acetaldehyde, and lactic acid. When plants face hypoxia, anaerobic respiration is strengthened to meet their energy needs, leading to accumulation of anaerobic products. Ethanol and lactate were enhanced and accumulated in cells, while energy charge was reduced in response to hypoxia and causes the acidification of cytoplasm, leading to death of plants [32].

Both plant ROS and unsaturated fatty acids when boosted resist adversity under hypoxia stress. The unsaturated components in lipids play an important role in removing ROS [33], as pointed out in a previous study [34]. This is positively consistent with the results of this study. With increasing stress, the content of linolenic acid was enhanced due to the perspective of gene expression. Indeed, arabidopsis ACBP3 participates in plant response to hypoxia by regulating VLCFA metabolism [35].

The phosphatidylglycerol (PG) is the site of oxidative electron transport of PS II, which is an important substance for chloroplast development. Since thylakoid membrane components were sensitive during stress, inactivation of the photosystem complex II reaction center reduced photosynthesis via affecting the absorption and conversion of light energy [36].

Lipid metabolites govern the perception and signal transduction in energy conversion, carbon storage, signal transduction, and regulation of hypoxia stress as a second messenger substance [37]. Phospholipase is induced and activated by flooding stress, and its hydrolysates activate proteases to maintain cell viability. Excessive production of PA caused by long-term water flooding will cause ROS explosion and cell death, damaging the integrity of cell membrane. Phospholipase was closely related to protein and gene regulation, which copes with stress and especially mediates the plant hypoxia signal chain [38]. This study reveals that lysophosphatidylcholine (LPC) is up-regulated in components of glycosphospholipids, inducing the production of ROS, which is mainly located in mitochondria and cytosol. Lyso PC is also up-regulated, affecting the deacylation/reacylation cycle and physiological metabolism. The mechanism of *Phyllostachys praecox* cell membrane responding to hypoxia was thus revealed.

Hypoxia reduces enzyme activity due to insufficient energy supply, further affecting fatty acid synthesis. The eicosanoids are mainly involved in energy storage and signal transduction, acting as structural components of cell membrane during stresses of hypoxia [39]. There are various changes in acyl coenzyme A with diverse chain length and saturation, but the changes of intracellular acyl coenzyme a pool are not obvious, as they activate the expression of hypoxia-related genes and start hypoxia signal transduction [37]. As previously demonstrated, there was an interaction between phosphatidylic acid and mitogen protein kinase during stresses of hypoxia, affecting the hypoxia response signal transduction. Another study also reported that during drought stress, lipids expression decreased steadily by 73.6%. In contrast, the prenol lipids class of lipids continued to drop, indicating that variable stress factors have various influence pathways on lipid components [40].

Compounds such as nucleic acids, fats, and steroids can also enhance plant resistance. Changes of metabolite levels during hypoxia stress were focused on metabolites of C and N metabolism as well as changes of organic acids and amino acids, while no change was observed in levels of sugar and sugar phosphate [41]. The content of proline was significantly improved with hypoxia stress. Pyrimidine nucleotide is one of the most basic components in cells, affecting the vast area of normal cell metabolism and playing a defensive role [42]. Nucleoside metabolism and membrane transport showed that metabolism was enhanced with reducing oxygen concentration, which suggests that nucleotide and membrane transport was up-regulated under hypoxia stress, actively responding to stress to adjust physiological activities to cope with stress. This illustrates that the plant metabolism actively adjusted its physiological changes to cope with stress.

Insufficient O_2_ leads to excessive electrons in cells and leaks out from inner membrane of mitochondria to produce reactive oxygen species (ROS). As a signal molecule, it activates the downstream signal pathway or causes oxidative rupture, resulting in cell damage [43]. At the same time, the intracellular osmotic pressure increases, and the cell water-absorption nuclear membrane breaks down, causing a series of metabolic disorders under hypoxia. All amino acid, carbohydrate, and lipid metabolism are generally the key metabolic pathways for plants to deal with stress factors [44]. This is consistent with the conclusion of this study.

As proven by the studies, amino acid metabolism was actively involved in stress [45]. Shingaki-Wells et al. reported that rice was involved in protein translation and antioxidant defense during hypoxia and up-regulated the expression of serine, glycine, and alanine biosynthesis from glyceraldehyde-3-phosphate or pyruvate [46]. Rocha et al. also revealed that alanine aminotransferase activity was improved and alanine accumulated when hypoxia was induced in soybean [47]. During reoxygenation, the transcription level and alanine content is decreased. This study observed that expressions of diverse amino acid metabolites was various. Malic acid was down-regulated, and l-2-Aminoadipic acid was up-regulated, indicating that dissimilar amino acid metabolites play different roles.

**Figure 6:**
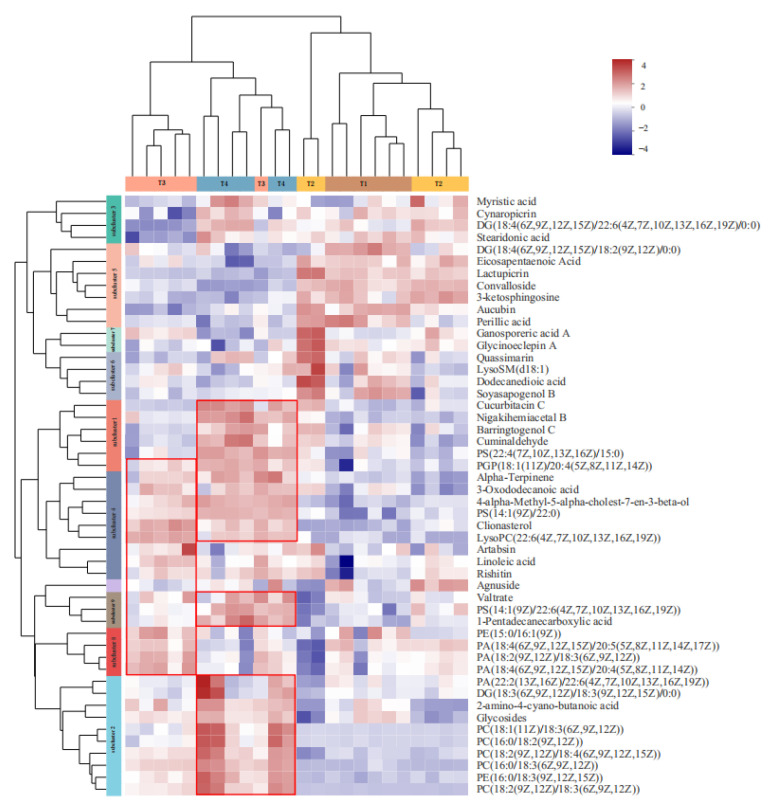
Cluster analysis of lipid metabolites among A (T1 vs. T2), B (T1 vs. T3), C (T1 vs. T4). (Each column in the figure represents a sample, and each row represents a metabolite. The colors in the figure represent the relative expression levels of metabolites in this group of samples. Cool colors are low). The left side is the dendrogram of metabolite clustering; the right side is the name of the metabolite, and the closer the two metabolite branches are, the closer their expression levels are. The top is the dendrogram of the sample clustering, and the two the closer the sample branches are, the closer the expression patterns of all metabolites in the two samples are, that is, the closer the metabolite expression trends are.

Carbohydrate metabolism was active and redistributed in response to stress [48], driving carbon distribution of plant organisms during stress [49]. Lipid metabolism can determine the structure and energy supply of plant cells [50], mediate signal transduction, and complete the cooperation between upstream and downstream substances and information [51]. We also observed 14 amino acid metabolic pathways. This finding suggests that glycospholipid metabolism is a key pathway in response to hypoxia stress, as the abundance further confirms that content of glycerol phospholipids (PC, PE, PA, etc.) was significantly improved during hypoxia stress (Figure 6). The content of glycerol lipids (DG) was also enhanced significantly, and LysoSM (d18:1), an intermediate of sphingolipid metabolism, was reduced. In addition, soyasapogenol B, eicosapentaenoic acid, lactupicirin, convaloside, 3-ketosphingosine, perilic acid, and aucubin decreased with the strengthening of stress.

Glycerol phospholipid metabolism was the central hub of the whole channel, and its metabolites were phospholipids. Glycerol triphosphate was an essential component in glycolysis and glycerol ester synthesis and metabolism but also is an important metabolite in the process of plant growth [52]. The glycerol triphosphate improved drought resistance of *Arabidopsis thaliana* via interfering with membrane lipid fluidity during expression of related genes [53]. The conversion of fatty acids to alkanes promotes the formation of plant cuticle wax [54], which was a metabolite involved in TCA cycle [55].

**Figure 7:**
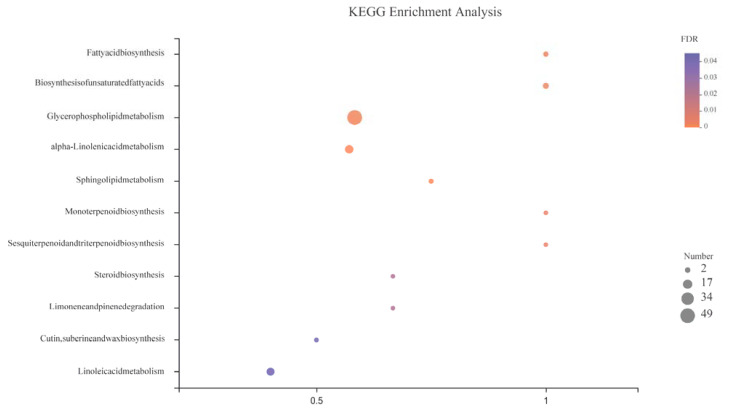
Metabolic pathways involving lipid metabolites in this study.

Lipids participate in other pathways to maintain physiological metabolism (Figure 7). It affects the biosynthesis of fatty acids, steroids, sesquiterpenes, and triterpenes through biosynthesis of unsaturated fatty acids and actively regulates the expression of linolenic and linoleic acid as well as sphingolipids to jointly maintain redox balance [13]. ROS [14], plant hormones [56], transcription factors [57], and protein kinases [58] jointly regulate the stress response of plants in response to hypoxia stress [59].

## 5. Conclusions

This study revealed that hypoxia stress significantly inhibited growth. The maximum reduction value in biomass was 42.8% with 2 mg/L treatment. The SPAD value was initially enhanced and then diminished with time during the whole culture cycle. The nutrient absorption and transport decreased significantly, as the root system activity and the nutrient content in stems and leaves per unit mass and were reduced during hypoxia stress. The contents of ethanol, acetaldehyde, and lactic acid was enhanced with decreasing oxygen concentration. The relative accumulation of metabolites of *Phyllostachys praecox* was improved to a certain extent or in a certain time, including the metabolism of organic acids, amino acids, and lipids, which were the main potential metabolic pathways to deal with hypoxia stress. The up-regulated expression of linolenic and linoleic acid as well as sphingolipids was influenced by lipids in response to hypoxia stress.

More importantly, we found that glycosphospholipid metabolism is the key pathway to response to hypoxia stress, which was demonstrated with significance (*p* < 0.05); lysoPC and PC in the metabolites of this metabolic pathway were significantly enhanced. Our investigation confirms the mechanism of *Phyllostachys praecox* cell membrane responding to hypoxia stress on molecular level.

This is conducive to finding targeted solutions to improve the productivity of *Phyllostachys praecox* to better optimize a mulching approach in the bamboo forest.

## Figures and Tables

**Table 1:** Effects of hypoxia stress on MDA of *Phyllostachys praecox*.

Treatments	0 d	3 d	5 d	7 d	12 d
8 mg/L	5.50 ± 0.32 a	5.02 ± 0.29 c	5.02 ± 0.41 d	4.7 ± 0.33 c	4.6 ± 0.13 d
6 mg/L	4.87 ± 0.37 a	5.42 ± 0.82 c	6.04 ± 0.49 c	6.87 ± 0.65 b	7.13 ± 0.39 c
4 mg/L	4.97 ± 0.24 a	6.42 ± 0.12 b	7.32 ± 0.44 b	11.03 ± 0.84 a	14.26 ± 1.04 b
2 mg/L	5.04 ± 0.69 a	7.98 ± 0.76 a	9.20 ± 0.91 a	12.85 ± 0.96 a	16.82 ± 1.26 a

Note: Different letters indicate significant difference between different treatments in the same period (*p* < 0.05).

**Table 2:** Effects of hypoxia stress on respiratory products concentrations of *Phyllostachys praecox* (μg/g).

Treatments	Ethanol	Acetaldehyde	Lactic Acid
8 mg/L	23.4 ± 2.3 d	52.1 ± 5.3 c	335.5 ± 19.4 d
6 mg/L	60.5 ± 5.7 c	96.9 ± 7.5 b	643.7 ± 63.5 c
4 mg/L	120.0 ± 10.5 b	93.1 ± 4.7 b	1126.8 ± 106.2 b
2 mg/L	243.0 ± 12.6 a	109.7 ± 10.3 a	1309.0 ± 125.5 a

Note: Different letters indicate significant difference between different treatments in the same period (*p* < 0.05).

**Table 3:** Screening of differential metabolome.

Mode	Total Number		
	A (T1 vs. T2)	B (T1 vs. T3)	C (T1 vs. T4)
pos	21 (210)	42 (282)	23 (162)
neg	20 (368)	18 (391)	15 (298)

## Data Availability

The data presented in this study are available on request from the corresponding author.
